# Environment sensing in spring-dispersed seeds of a winter annual Arabidopsis influences the regulation of dormancy to align germination potential with seasonal changes

**DOI:** 10.1111/nph.12694

**Published:** 2014-01-21

**Authors:** Steven Footitt, Heather A Clay, Katherine Dent, William E Finch-Savage

**Affiliations:** 1 School of Life Sciences, University of WarwickWellesbourne Campus, Warwickshire, CV35 9EF, UK

**Keywords:** Arabidopsis, *DOG1*, dormancy cycling, hormone signalling, *MOTHER OF FLOWERING TIME*, nitrate, soil seed bank, temperature

## Abstract

1. Seed dormancy cycling plays a crucial role in the lifecycle timing of many plants. Little is known of how the seeds respond to the soil seed bank environment following dispersal in spring into the short-term seed bank before seedling emergence in autumn.
2. Seeds of the winter annual Arabidopsis ecotype Cvi were buried in field soils in spring and recovered monthly until autumn and their molecular eco-physiological responses were recorded.
3. *DOG1* expression is initially low and then increases as dormancy increases. *MFT* expression is negatively correlated with germination potential. Abscisic acid (ABA) and gibberellin (GA) signalling responds rapidly following burial and adjusts to the seasonal change in soil temperature. Collectively these changes align germination potential with the optimum climate space for seedling emergence.
4. Seeds naturally dispersed to the soil in spring enter a shallow dormancy cycle dominated by spatial sensing that adjusts germination potential to the maximum when soil environment is most favourable for germination and seedling emergence upon soil disturbance. This behaviour differs subtly from that of seeds overwintered in the soil seed bank to spread the period of potential germination in the seed population (existing seed bank and newly dispersed). As soil temperature declines in autumn, deep dormancy is re-imposed as seeds become part of the persistent seed bank.

## Introduction

At the molecular level a range of mechanisms and signalling networks have been identified that regulate seed dormancy under laboratory conditions (Finch-Savage & Leubner-Metzger, 2006; Finkelstein *et al*., 2008; Nambara *et al*., 2010; Graeber *et al*., 2012). Manipulating seed dormancy states in the laboratory has further increased our understanding of how these molecular pathways and physiological components operate (Cadman *et al*., 2006; Finch-Savage *et al*., 2007). These regulatory mechanisms are dominated by a dynamic balance between the hormones abscisic acid (ABA) and gibberellins (GA), and the cohorts of genes that regulate their metabolism, perception and sensitivity via signalling networks considered central to dormancy and the control of germination completion (radical emergence through the seed coat; Finch-Savage & Leubner-Metzger, 2006; Nambara *et al*., 2010; Bassel *et al*., 2011; Morris *et al*., 2011; Dekkers *et al*., 2013).

However, a greater understanding of the annual pattern of changing dormancy status (dormancy cycling) in the natural environment is required to advance our understanding of soil seed bank dynamics in contexts such as the emergence timing of weed species for modelling weed/crop competition dynamics (Batlla *et al*., 2004), and the maintenance of species diversity and restoration of natural populations, especially in response to future climate change (Jump *et al*., 2009; Ooi *et al*., 2009). Eco-physiological studies on dormancy cycling in several species under field conditions have revealed seasonal changes in seed sensitivity to temperature, light, nitrate and GA leading to large differences in the germination response from month to month (Bouwmeester & Karssen, 1993a,b; Derkx & Karssen, 1993, 1994). This indicates that seeds are able to sense changes in the local soil seed bank environment and rapidly adjust their dormancy status in response.

In Arabidopsis, winter annual ecotypes such as Cape Verdi Isle (Cvi) normally germinate and establish in the autumn and overwinter as rosettes, before flowering and shedding seed in spring (Baskin & Baskin, 1972; Donohue, 2009; Footitt *et al*., 2011). Whereas, in the Arabidopsis summer annual Burren (Bur) seeds over winter in the soil before emerging in spring to flower over the summer period (Ratcliffe, 1976; Footitt *et al*., 2013). Before dispersal from the parent plant, the depth of seed dormancy is strongly affected by the maternal environment with nitrate, temperature and photoperiod having strong effects on dormancy (Alboresi *et al*., 2005; Donohue, 2009; Kendall & Penfield, 2012). Once dispersed seeds remain responsive at a molecular level in both the imbibed and dry state as dormancy changes (Rajjou *et al*., 2004; Cadman *et al*., 2006; Finch-Savage *et al*., 2007; Carrera *et al*., 2008; Arc *et al*., 2012).

In previous work we applied a molecular eco-physiological approach to the study of dormancy cycling in the field with two contrasting Arabidopsis ecotypes having winter and summer annual behaviour (Footitt *et al*., 2011, 2013). Seeds of both ecotypes were placed in soil in autumn to investigate the behaviour of seeds persisting in the seed bank. In this way we were able to show the temporal coordination of the major signalling networks that regulate seed dormancy in an ecological context. This highlighted that seeds in the seed bank are capable of adjusting the depth of dormancy through temporal sensing (identifying the correct season and climate space for emergence) and spatial sensing (identifying signals indicating suitable conditions to terminate dormancy and complete germination). Dormancy and the expression of dormancy-related genes were highly sensitive to the soil environment and molecular physiological changes could be equated to changes in sensitivity to soil temperature history, nitrate, light and GA. This shows dormancy to be a continuum with layers of dormancy being progressively removed by environmental signals until only light is required, in the absence of which seeds remain dormant and enter into another dormancy cycle as the seasons change (Footitt *et al*., 2011, 2013; Finch-Savage & Footitt, 2012).

Here we present an investigation of the response of dormant Cvi seeds buried in the soil seed bank at their natural dispersal time in spring. Following this spring dispersal into warm soils, seeds rapidly align their dormancy cycle to the prevailing soil environment with a rapid transition into the spatial sensing phase. This contrasts with ungerminated seeds remaining in the soil seed bank at the end of the season in the cold soils of autumn. These overwintering seeds enter a deep dormancy phase of temporal sensing before re-entering the shallow dormancy spatial sensing phase (Footitt *et al*., 2011). Following spring dispersal of Cvi, spatial sensing dominates resulting in rapid changes in gene expression compared to that seen in autumn buried Cvi. Differences in expression of the gene *MOTHER OF FLOWERING TIME* (*MFT*) in spring dispersed and overwintered (soil seed bank) seeds indicate an adaptive response to the spread of germination timing in the seed population. This and the role of *DOG1* are discussed in the context of dormancy cycling behaviour in short-term and persistent seed banks of both winter and summer annual ecotypes.

## Materials and Methods

### Seed production

Plants of *Arabidopsis thaliana* (L.) Heynh, ecotype Cape Verdi Isle (Cvi) were grown from seed (sown February 2007) in a temperature-controlled glasshouse. Seeds were harvested at maturity (May 2007), processed and stored at −80°C to minimise physiological change. The seed lot produced was used in both this study and that previously reported for seeds overwintered in the soil seed bank (Footitt *et al*., 2011).

### Seed burial

Seeds were dressed with Metalaxyl (Hockley International, Manchester, UK) at 1 g active fungicide kg seeds^−1^ and dispersed in Ballotini balls (100–250 μm diameter; Potters Ballotini Ltd, Barnsley, UK) of the same particle size as the sand in the sandy loam soil in the field trial area. Seeds were dispersed at a density of 40 seeds g^−1^ of Ballotini balls to reduce seed mortality, placed in 125-μm mesh nylon bags (Clarcor-UK, Warrington, UK) and sealed with a WeLoc® bag clip (size PA110; Weloc Scandinavia Direct Ltd, Crowborough, UK). Seed bags were buried at a depth of 5 cm in a randomised plot design on 9 May 2008. SM200 soil moisture sensors (Delta-T Devices Ltd, UK) and Thermistore temperature probes (Betatherm, Ireland) linked to a data logger (Delta-T Devices Ltd, Cambridge, UK) recorded soil moisture and temperature at seed depth in dummy bags (Footitt & Finch-Savage, 2011; Footitt *et al*., 2011).

### Seed recovery

Samples for molecular analysis were recovered from the field in the dark. A light-proof box with sealed arm holes at the top was placed over the burial site and the base sealed with soil to exclude light. The samples were then exhumed and placed in a laminated foil bag (Moore and Buckle, St Helens, UK) sealed with a WeLoc® PA150 clip. In the laboratory under a green safe light seed bags were quickly rinsed in cold water to removes excess soil, the contents emptied into 50 ml centrifuge tubes and seeds immediately separated from the Ballotini balls in cold water. Ballotini balls and seeds were allowed to settle, and then washed three times. Tubes were gently shaken to move seeds over the Ballotini balls to the tube walls where they were transferred with a pastette (3 ml; Jencons Ltd, Bath, UK) to 2 ml Eppendorf tubes, excess water was removed and tubes immediately frozen in liquid nitrogen and stored at −80°C. Seeds for physiological analysis were separated from Ballotini balls in the light in a similar fashion as above and immediately used for dormancy testing (Footitt & Finch-Savage, 2011).

### Dormancy evaluation

Seeds recovered for physiological analysis were surface-sterilized in a 0.125% sodium hypochlorite solution (Household bleach (5% sodium hypochlorite) diluted to 2.5%) for 5 min then washed three times in water. Using an Ultipette™ BARKY CP-100 tips (Barkey Instruments International, Folkstone, UK) 40 seeds were plated out in triplicate plates (124 × 88 × 22 mm; Stewart Plastics Ltd, Croyden, UK). Each plate contained two sheets of Whatman 3MM chromatography paper (Scientific Laboratory Supplies Ltd, Nottingham, UK) and 8 ml of the appropriate solution. Plates were placed in sealable freezer bags and incubated at 20°C under continuous light and germination scored at 2–3 d intervals for up to 28 d. Nitrate sensitivity was tested by incubating seeds in 10 mM KNO_3_ as above. Gibberellin (GA) sensitivity was tested by exposing seeds to 5–250 μM GA4 + 7 in 1.7 mM citric acid/3.3 mM K_2_HPO_4_ buffer at pH 5.0, as above.

Thermodormancy was tested on water as above at 5, 10, 15, 20 and 25°C. Seed viability was also tested at 20°C using 100 μM GA4 + 7/50 μM Fluridone (Apollo Scientific, Stockport, UK) in citrate/phosphate buffer (pH 5.0). Depth of dormancy was determined by dry after-ripening seeds at 20°C in the dark at an equilibrium moisture content of 55% relative humidity. Loss of dormancy was evaluated by periodic germination testing on water at 20°C in the light. From the resulting after-ripening curves the depth of dormancy was determined as days to 50% germination (AR50).

### Quantitative PCR of genes associated with dormancy cycling

In order to investigate the role of hormone signalling during changes in thermodormancy and germination potential seen in the field we determined the expression levels of key genes in the synthesis of abscisic acid (ABA) and GA and their respective signalling pathways. The gene family members selected exhibited distinct seed expression patterns in our previous laboratory and field studies of dormancy cycling (Cadman *et al*., 2006; Finch-Savage *et al*., 2007; Footitt *et al*., 2011, 2013). Genes chosen were those involved in ABA and GA biosynthesis (*NCED6*, *GA3ox1*) and catabolism (*CYP707A2*, *GA2ox2*), ABA signalling (*PYR1*, *PYL7*, *ABI2, SnrK2.1* & *2.4*, *ABI3*, *ABI4* & *ABI5*), the ABA signalling regulator *MFT*, GA signalling (*GID1A*, *RGL2*, *RGA2*(*GAI*)), members of the Phytochrome Interacting Factor (PIF) family (*PIL5* and *SPT*), and the dormancy associated *DOG1*. The putative involvement of these genes in the regulation of dormancy is summarised in Footitt *et al*. (2011). Genes involved in nitrate sensing and processing, the nitrate transporter *NRT1* and the reductase *NR1*, were also analysed.

RNA was extracted using nuclease-free reagents as described in Footitt *et al*. (2013). RNA quality was then determined spectrophotometrically (Nanodrop, Wilmington, DE, USA) and treated with RNase-free DNase I (Roche Diagnostics, Burgess Hill, UK) to remove genomic DNA. Synthesis of cDNA was performed on 2 μg total RNA using SuperScript™ II Reverse Transcriptase (Invitrogen) and random pentadecamer primers (Invitrogen) following the manufacturers’ instructions. The qRT-PCR were performed in 384-well plates with a LightCycler® 480 Real-Time PCR instrument (Roche Diagnostics) using the LightCycler® 480 SYBR Green I Master kit (Roche Diagnostics). The gene-specific primer pairs used are listed in Supporting Information Table S1. The reference gene *At4g34270* (Tip 41-like) was used for normalization of the data due to its stability of expression in seed dormancy microarray data (Cadman *et al*., 2006; Finch-Savage *et al*., 2007) where it has a low-fold change in expression and coefficient of variation across 11 different hydrated dormancy states when compared with others evaluated as reference genes in plants (Remans *et al*., 2008). It was also identified as a highly stable reference gene for use in expression analysis by QPCR in Arabidopsis seeds (Dekkers *et al*., 2012). Reactions were performed in triplicate and contained 5 μl of SYBR Green I Master, 2 μl PCR-grade water, 1 μl of 10 mM forward and reverse primers, and 1 μl cDNA (diluted 1 : 25) in a final volume of 10 μl. PCR were carried out under the following conditions: one cycle at 95°C for 10 min followed by 50 cycles at 95°C for 30 s, 65°C for 30 s, and 72 C for 30 s. Data were analysed using Light Cycler® 480 software (v1.5; Roche Diagnostics).

## Results

### Changing dormancy levels following spring burial

**Figure 1:**
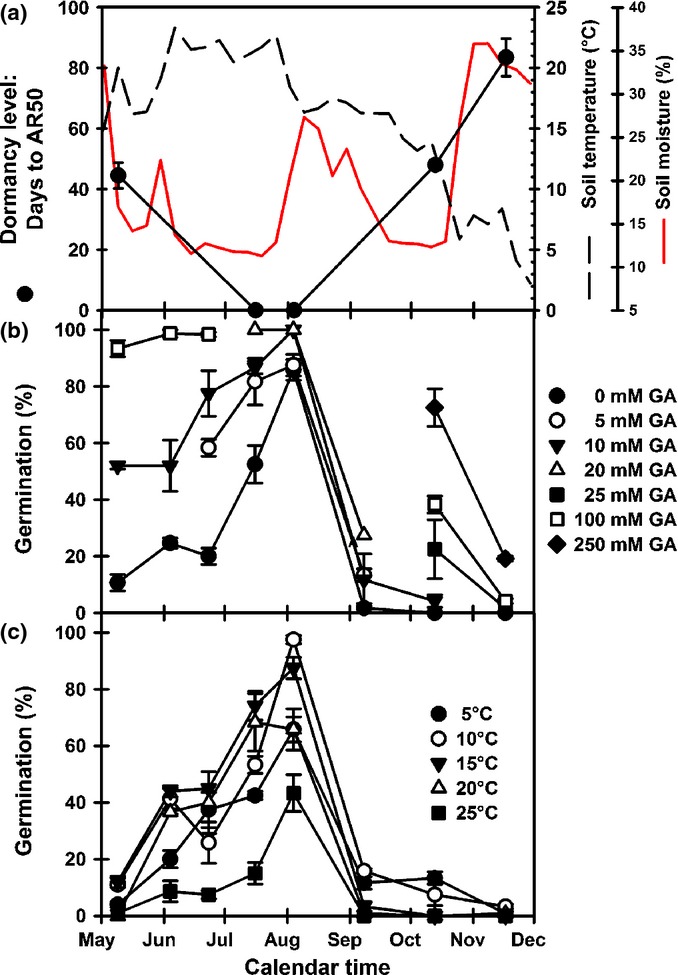
Seasonal changes in dormancy cycling in spring buried Arabidopsis (Cvi). (a) Changes in dormancy level (AR50), and soil temperature and moisture content measured at seed depth (5 cm). (b) Changing sensitivity of seeds recovered from the field to gibberellins (GA) at 20°C in the light. (c) Changing thermodormancy in seeds recovered from the field. Following recovery, seeds where incubated in the light at 5–25°C. AR50, time to 50% after-ripening. Error bars, ± SEM, *n* = 3.

Seeds of the winter annual Arabidopsis ecotype Cvi germinate and emerge in the autumn, overwintering as rosettes that flower in early spring. Seed shedding from these overwintered plants in the UK is seen from late April to late May depending on winter/spring temperatures (S. Footitt and W. E. Finch-Savage unpublished). To evaluate the behaviour of the short-lived seed bank represented by this spring to autumn dormancy cycle, primary dormant seeds of the Cvi ecotype were buried in the field in late spring to mimic the natural time of seed dispersal. Before burial in spring seeds were primary dormant, with the depth of dormancy as estimated by the time to 50% after-ripening being *c*. 44 d (AR50). Following burial, AR50 rapidly declined and by July seed dormancy was at its lowest with only light required to remove the final block to germination (Fig.1). By September, AR50 values increased as dormancy again increased reaching an AR50 value of 83 d when the experiment concluded in November 2008. Changes in AR50 were inversely related to soil temperature (Fig.1a).

### Germination potential rapidly aligns to the soil environment

**Figure 2:**
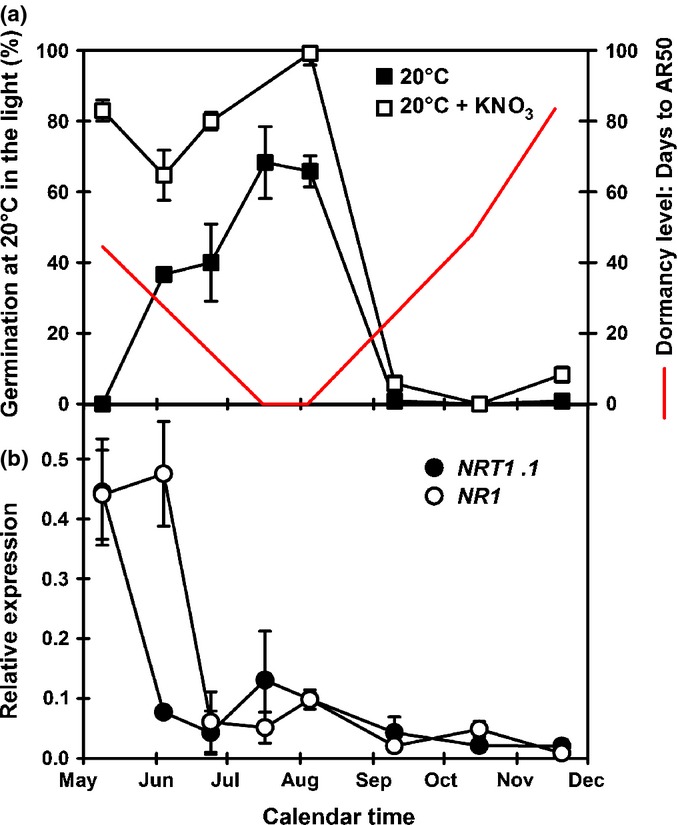
Nitrate sensitivity during dormancy cycling of Arabidopsis (Cvi) in the field. (a) Germination in the light at 20°C ± 10 mM KNO_3_ in relation to changing dormancy level (AR50). (b) Expression of nitrate transporter (*NRT1.1*) and nitrate reductase (*NR1*). AR50, time to 50% after-ripening. Error bars, ± SEM, *n* = 3.

The rapid decline in dormancy on burial was reflected in the GA sensitivity of recovered seeds, which increased up until August (Fig.1b). After which, it rapidly declined as dormancy increased to a point where even 250 μM GA gave a minimal response. The magnitude of thermodormancy in seed recovered from the field (Fig.1c) also reflected this changing environmental sensitivity. In general, high-temperature thermodormancy declined to its lowest by August as soil temperature increased. At this time the temperature range permitting germination was at its widest and when this coincided with ambient soil temperature (thermal germination window), the onset of seedling emergence was observed in the field following soil disturbance and exposure to light (Footitt *et al*., 2011). Germination at 25°C was always lowest, but interestingly the relative levels of germination at other temperatures were not consistent and the reason for this is not obvious. Following maximum germination at all temperatures in August, the germination window subsequently closed and thermodormancy rapidly increased as dormancy increased in response to decreasing soil temperature. By September, seeds were almost completely unresponsive to any temperature tested. Over the same period, nitrate sensitivity was maximal during August, after which sensitivity was lost (Fig.2a). At the molecular level, expression of *NRT1* and *NR1* were high before burial, *NRT1* expression declined on burial closely followed by *NR1* (Fig.2b).

### Gene expression differs in newly dispersed seeds compared to seeds overwintered in the seed bank

We looked for similarities in gene expression during dormancy cycling between seeds overwintered in the seed bank from autumn 2007 (Footitt *et al*., 2011) and seeds of the same seed lot newly dispersed to the seed bank in spring 2008 (this study). To do this we conducted correlation analysis of gene expression values in the period where the two time-courses overlapped (June–October 2008) and therefore the soil environment was the same in both studies. Only four genes showed significant correlations in their expression patterns; *DOG1* (*r* = 0.96, *P* < 0.01), *ABI5* (*r* = 0.91, *P* < 0.05), *GA2ox2* (*r* = 0.92, *P* < 0.05) and *NCED6* (*r* = 0.85, *P* < 0.1). This indicates that the response of seeds experiencing shallow dormancy in the summer following spring dispersal differed markedly from those of the same population that had first experienced cycling into deep dormancy during overwintering.

**Figure 3:**
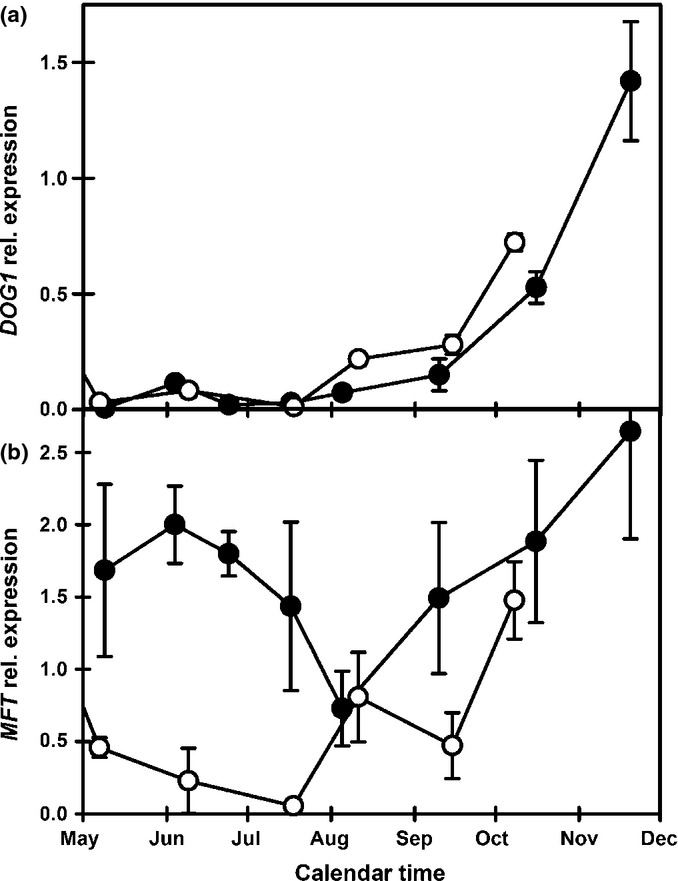
Comparison of *DOG1* and *MFT* expression in Arabidopsis (Cvi) seeds buried in the field in Autumn 2007 (open circles) and Spring 2008 (closed circles). (a) Changes in *DOG1* expression from May to December 2008. (b) Changes in *MFT* expression from May to December 2008. Error bars, ± SEM, *n* = 3.

The dormancy-associated gene *DELAY OF GERMINATION1* (*DOG1*) had a similar pattern in both overwintered and spring-dispersed seeds (Fig.3a) and is consistently correlated with soil temperature (Table S2). However, the gene, *MOTHER OF FLOWERING TIME* (*MFT*) which is significantly correlated (*P* < 0.01) with dormancy, soil temperature and *DOG1* in seeds overwintered in the seed bank (Footitt *et al*., 2011) has correlations with greatly reduced significance (*P* > 0.05 < 0.1; Table S2) and a different expression pattern in seeds dispersed in spring (Fig.3b).

### Changes in hormone signalling

**Figure 4:**
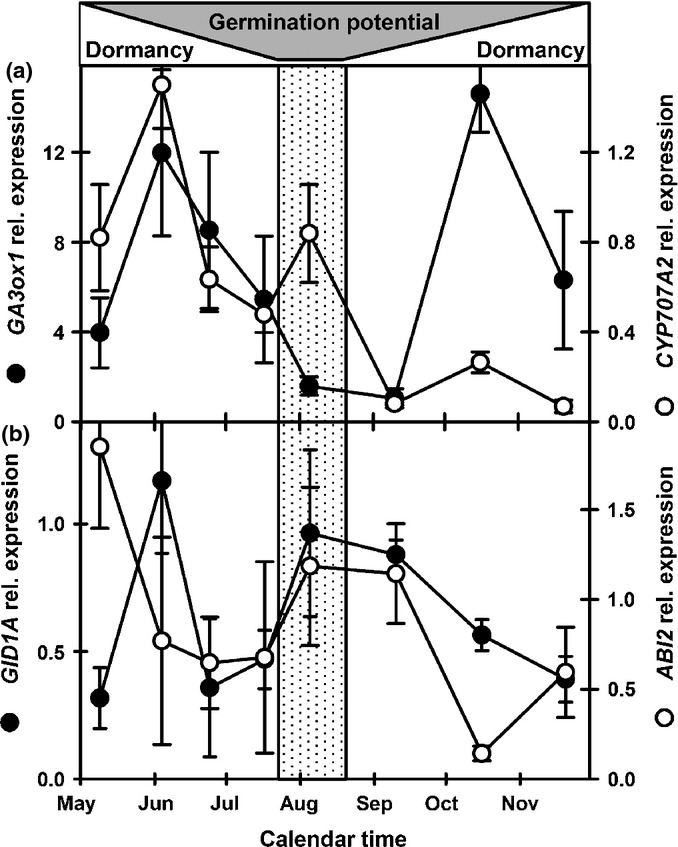
Comparison of gene expression involved in increasing GA sensitivity in Arabidopsis (Cvi). (a) Expression of *GA3ox1* (gibberellin (GA) biosynthesis) and *CYP707A2* (abscisic acid (ABA) catabolism). (b) Expression of *GID1A* (GA receptor) and *ABI2* (repressor of ABA signalling). Shaded area indicates changing germination potential in relation to dormancy. Vertical shaded panel represents the period of maximum germination potential. Error bars, ± SEM, *n* = 3.

**Figure 5:**
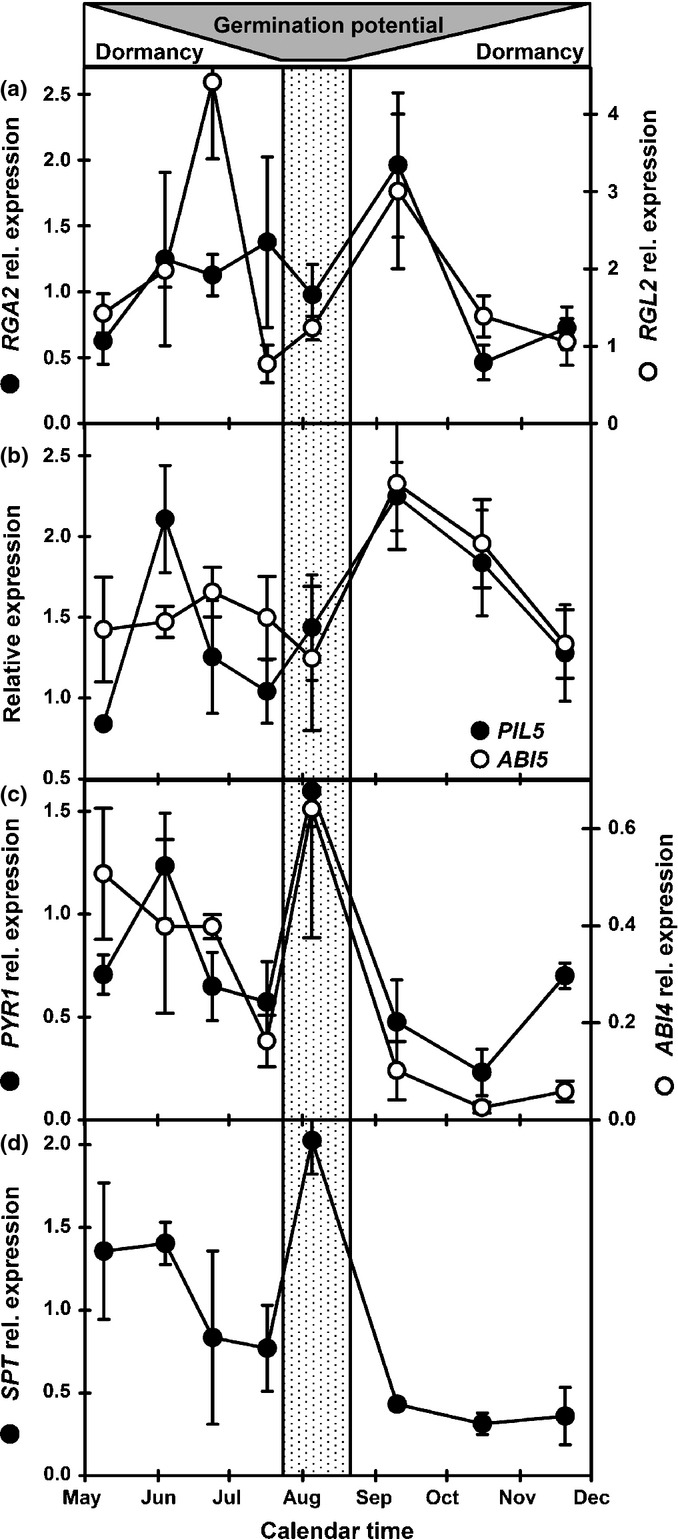
Comparison of gene expression involved in suppression of germination potential in Arabidopsis (Cvi). (a) Expression of *RGA2* and *RGL2* (DELLAs – germination repressors). (b) Expression of *PIL5* (bHLH transcription factor of the PIF family – germination repressor) and *ABI5* (abscisic acid (ABA) induced germination repressor). (c) Expression of *PYR1* (ABA receptor) and *ABI4* (control of energy utilisation). (d) Expression of *SPT* (bHLH transcription factor of the PIF family – germination repressor). Horizontal shaded area indicates changing germination potential in relation to dormancy. Vertical shaded panel represents the period of maximum germination potential. Error bars, ± SEM, *n* = 3.

As dormancy declined the expression of *CYP707A2* (ABA catabolism) and *GA3ox1* (GA biosynthesis) increased transiently consistent with overwintering in the seed bank (Footitt *et al*., 2011), then decreased to a low level with a second transient peak of *GA3ox1* as dormancy increased in autumn (Fig.4a). Like *CYP707A2* and *GA3ox1*, *GID1A* (GA receptor) showed an early peak in expression. Subsequently both *GID1A* and *ABI2* (repressor of ABA signalling) had broad peaks of expression from the peak in germination potential (August) to the re-imposition of dormancy in September (Fig.4b). In September, the repressors of GA signalling *PIL5* and the DELLA genes *RGA2* and *RGL2* all peaked, as did the ABA-induced repressor of germination potential *ABI5* (Fig.5a,b).

The ABA-signalling genes *PYR1* (ABA receptor), *ABI4* (repressor of germination potential; Fig.5c) and *SPT* –a transcription factor of the Phytochrome interacting factor family (Fig.5d) – have large peaks in expression (with a smaller peak for *CYP707A2*) when seeds are most sensitive to temperature, light and nitrate in August (Fig.1c).

**Figure 6:**
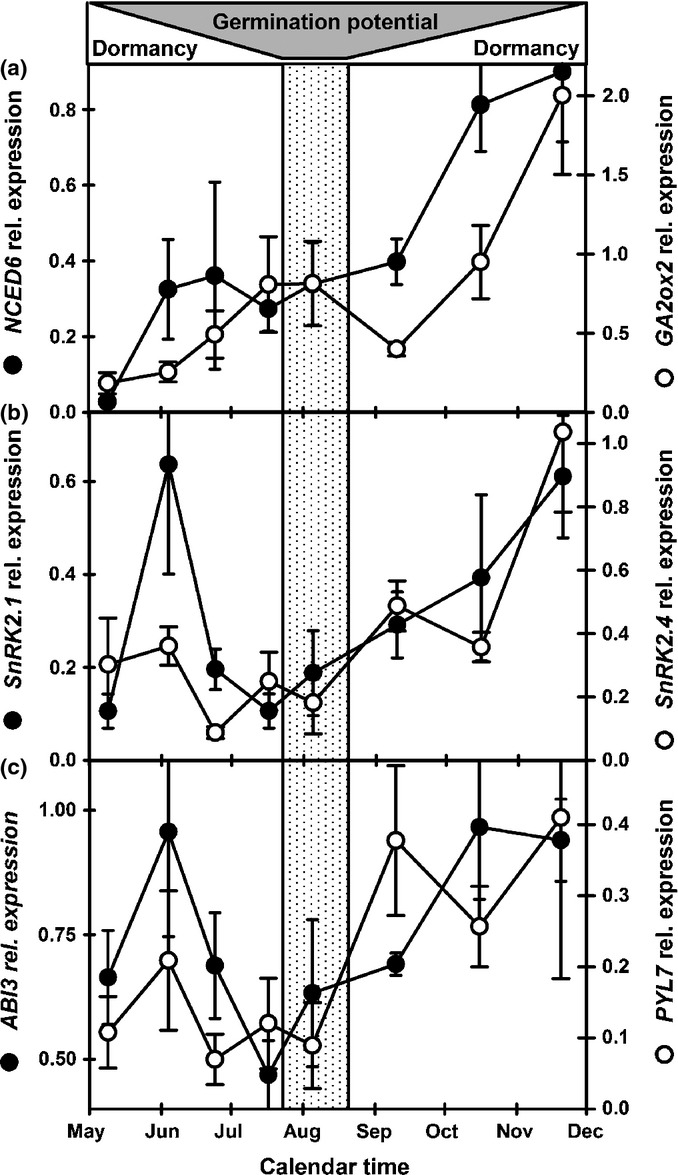
Gene expression involved in ABA signalling and dormancy in Arabidopsis (Cvi). (a) Expression of *GA2ox2* (gibberellin catabolism) and *NCED6* (abscisic acid (ABA) biosynthesis). (b) Expression of *SnRK2.1* and *SnRK2.4* (positive regulators of ABA signalling). (c) Expression of *ABI3* (dormancy-associated transcription factor) and *PYL7* (ABA receptor). Shaded area indicates changing germination potential in relation to dormancy. Vertical shaded panel represents the period of maximum germination potential. Error bars, ± SEM, *n* = 3.

In late spring and summer *NCED6* (ABA synthesis) and *GA2ox2* (GA catabolism) expression is low, increasing as dormancy subsequently increases from late August (Fig.6a). This is coincident with increased expression of *PYL7* (ABA receptor), the SNF1-related protein kinases *SnrK 2.1* & *2.4* (positive regulators of ABA signalling that link ABA receptor activity to transcriptional regulators; Nambara *et al*., 2010) and the transcriptional regulator *ABI3* (Fig.6b,c). This pattern is consistent with that of *DOG1* with all negatively correlated to soil temperature (Table S2). Interestingly there is also a peak of *ABI3* and *SnRK2.1* (dormancy promoting) expression immediately after sowing coincident with the transient initial increase in *CYP707A2, GID1A* and *GA3ox1* (dormancy relieving) expression.

## Discussion

We have used the Arabidopsis accession Cvi because it has become an accepted model for dormancy studies and we have previously shown that it exhibits the lifecycle of a winter annual in UK soil conditions (Footitt *et al*., 2011). Nevertheless having been collected in the Cape Verde Islands it may not be naturally adapted to these conditions. Here we show the response of primary dormant seeds of this ecotype buried in the soil seed bank in spring, the natural shedding and dispersal season for winter annuals (Baskin & Baskin, 1972; Donohue, 2009). The physiological changes in the dormancy status of these seeds in response to warm spring soil are broadly similar to those reported for the same seed lot overwintered following burial in autumn (Footitt *et al*., 2011). However, changes in gene expression identify more subtle differences in the interpretation of environmental signals linked to spatial sensing in these two cohorts of seeds. Understanding the behaviour of the whole seed population (existing seed bank and newly dispersed) has wide significance because it determines the timing and spread of new entrants to natural plant communities or crop competing weed populations.

### Change in dormancy status

Upon burial in spring, dormancy immediately decreased. At this time soil temperature was increasing and soil moisture content was between 11% and 17%. Under these dry conditions dry after ripening may have played a role in these shallow dormant seeds. By contrast, the deep dormancy of over-wintered seeds was lost more rapidly in spring than could be accounted for by dry after-ripening and appeared to have occurred in the imbibed state (Footitt *et al*., 2011). However, high-temperature thermodormancy decreased and GA sensitivity increased in a broadly similar fashion in both seed cohorts, revealing dormancy to be lowest around August. In both cohorts there was a subsequent rapid induction of deep dormancy in the month following peak germination potential. Overall these responses are consistent with other Arabidopsis field and glasshouse studies (Baskin & Baskin, 1972, 1983; Derkx & Karssen, 1994; Footitt *et al*., 2011, 2013). We consider below the regulation of germination potential at a molecular level as suggested by seasonal changes in the transcription of key genes with known functions in the control of dormancy.

### The role of *DOG1*

**Figure 7:**
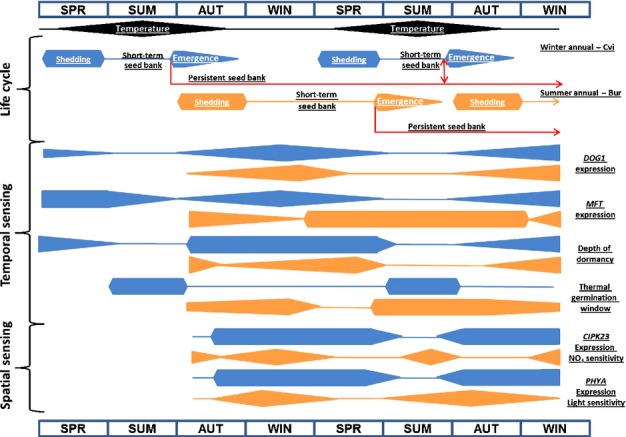
Seed responses (molecular and physiological) to environmental sensing (temporal and spatial) drive progress through the annual dormancy cycle to time the key lifecycle transition from seed to seedling (germination) in Arabidopsis. The schematic summarises data from the present study, Footitt *et al*. (2011, 2011) and illustrates how subtle differences in the coordination of these responses can result in winter and summer annual lifecycles of Arabidopsis ecotypes. These studies have also shown how hormone metabolism and signalling networks operate to put into effect these responses to the soil environment. In the schematic the winter annual ecotype Cvi is represented by the blue bars and the summer annual ecotype Bur by the orange bars. The height of each bar indicates the amplitude of the response measured across the seasons. Temperature represents the annual fluctuation in soil temperature at seed depth. Seed shedding times and emergence timing are based on the field observations. The short-term seed bank is made up of those seeds that are shed from the mother plant, enter the soil, lose dormancy and germinate in the soil in one dormancy cycle so contributing to the next sequential generation. The persistent seed bank represents seeds that pass through more than one annual dormancy cycle thus dispersing seeds in time. Temporal sensing shows the major changes in molecular (*DOG1* & *MFT*) and physiological markers for dormancy. Depth of dormancy is related to the time to 50% after-ripening (AR50; Cvi) and *Ψb* (base water potential; Bur) in seeds. The thermal germination window represents that period when as a result of declining thermodormancy the thermal base line for germination and soil temperature overlap, so permitting germination and seedling emergence if other environmental requirements are met (water, light, nitrate). Spatial sensing shows when seeds exhibit increased germination potential as reflected by sensitivity to environmental signals such as light (*PHYA*) and nitrate (*CIPK23*), which inform the seed as to its position in relation to the soil surface and existing vegetation.

The *DOG1* locus has the strongest dormancy association in QTL analyses (Bentsink *et al*., 2006). Its expression is not directly associated with [ABA], but may enhance ABA sensitivity (Footitt *et al*., 2011). In the field, expression of *DOG1* greatly increased as soil temperature decreased in the autumn in both spring and autumn burials of Cvi and in the summer annual Bur buried in autumn (Footitt *et al*., 2011, 2013; Fig.7). In Cvi (winter annual) this change is positively correlated with dormancy, but it is not in Bur (summer annual) indicating subtle differences between ecotypes that readily cycle into deep dormancy (Cvi) compared to those adapted to a shallow dormancy cycle (Bur). This is consistent with the idea that accumulation and retention of DOG1 protein via temperature sensing is directly related to the depth and persistence of dormancy (Nakabayashi *et al*., 2012; Footitt *et al*., 2013). Although experimental verification will be required, we propose that *DOG1* is part of a thermal sensing mechanism that measures the passage of time (temporal sensing) with the accumulation of DOG1 serving to represent accumulated thermal time to regulate the depth and persistence of dormancy.

### The role of *MFT*

MFT is a member of the phosphatidylethanolamine-binding protein (PEBP) family, which includes flowering time regulators FLOWERING LOCUS T (FT) and TERMINAL FLOWER 1 (TFL1). The *MFT* gene is downregulated by ABI3 and upregulated by the DELLAs, RGA2 and RGL2, and by ABI5, the latter being in turn downregulated by MFT (Xi *et al*., 2010). It has therefore been assigned the role of negatively regulating ABA signalling to promote embryo growth during germination. However, this is difficult to reconcile with our field observations in Cvi (overwintered and newly sown), where *MFT* expression was negatively correlated to germination potential (*P* < 0.05 at 5 and 10°C) and so increased with dormancy. However, despite these correlations there was, unlike *DOG1*, a significant difference in gene expression in May–August between the overwintered and newly dispersed seeds (Figs7). In overwintered seeds *MFT* expression declined to low levels by May, but was relatively high in the newly dispersed seeds and initially remained high before declining to its lowest level in August (Fig.7). These differences may change with different soil conditions and the resulting germination timing differences in the two cohorts may contribute to bet hedging for the population as a whole.

The subsequent increase in *MFT* expression with declining soil temperature is consistent with observations in wheat where *MFT* expression increased > 10-fold in embryos matured at 13°C compared to 25°C (Nakamura *et al*., 2011). Cold conditions lead to increased dormancy in both wheat (Nakamura *et al*., 2011) and Cvi seeds (Fig.1; Footitt *et al*., 2011). However, in the low dormancy summer annual ecotype Bur, which naturally enters the cold soil seed bank in autumn, *MFT* and soil temperature are positively correlated (Footitt *et al*., 2013). Furthermore, *MFT* expression is closely associated with high germination potential and positively correlated with the genes involved in spatial sensing in Bur (data S1 in Footitt *et al*., 2013; Fig.7). This is consistent with the suggestion that *MFT* has a duel role in promoting both primary dormancy and germination potential in after-ripened seeds (Vaistij *et al*., 2013) and may be part of a temperature sensitive signalling system (Nakamura *et al*., 2011).

### Control of germination potential following dispersal

From spring sowing to maximum germination potential in August seeds were more nitrate sensitive than overwintered seeds in which sensitivity had previously been reduced by exposure to low temperatures (Footitt *et al*., 2011). Nitrate induces expression of the ABA catabolism gene, *CYP707A2* (Matakiadis *et al*., 2009). However, seed nitrate is rapidly lost in the soil (Derkx & Karssen, 1993) and nitrate uptake from the soil would be required by seeds to enhance ABA catabolism via the action of CYP707A2 (Matakiadis *et al*., 2009). A reduction in [ABA] in conjunction with the observed increase in both *GA3ox1* (GA biosynthesis) and *GID1A* (GA receptor) expression results in the increased germination potential upon exposure to light in the spatial sensing phase of the dormancy cycle that removes the final block to germination. Of the three members of the *GID* receptor family, GA signalling via *GID1A* & *C* is required for germination in Arabidopsis with only *GID1A* exhibiting a strong expression pattern during dormancy cycling in the laboratory (see dataset S2 in Footitt *et al*., 2011) (Cadman *et al*., 2006; Voegele *et al*., 2011). In both the newly dispersed and overwintered seeds *GID1A* and *ABI2* expression peaked when GA sensitivity was greatest (Figs5; Footitt *et al*., 2011). This in conjunction with increased *GA3ox1* expression in the light (Cadman *et al*., 2006) explains the nitrate enhancement of germination in the light when dormancy levels are low (Hilhorst & Karssen, 1988). Interestingly the initial transient *GID1A* peak in June is matched by the SNF1-related protein kinase, *SnrK 2.1* which may antagonise GA sensitivity at this point. This initial transient increase in the expression of genes linked to both dormancy promotion (*ABI3* and *SnRK2.1*) and dormancy relief (*CYP707A2*, *GID1A* and *GA3ox1*) following spring sowing suggest that seed behaviour is not fully determined during seed development, but that it also relies upon initial sensing of the environment following dispersal.

### Germination repression: the role of DELLAs

Countering the increasing potential to germinate in the soil, are a series of genes that repress germination potential in the absence of light. The DELLA family are major repressors of germination potential and GA signalling (Daviere *et al*., 2008). Here the expression of *RGA2* and *RGL2* were highest immediately before and after the largest changes in germination potential. *RGA2* had a single peak in both newly dispersed and overwintered seeds, whereas *RGL2* had two peaks in the former, but only one in the latter. This indicates that the role of DELLAs in spatial sensing may be more responsive to the environment in those seeds dispersed to the seed bank in the spring. At the same time DELLAs also depress metabolic activity in seeds by repressing genes related to carbohydrate, lipid and protein metabolism, potentially acting in concert with *ABI4* (Cao *et al*., 2006; Penfield *et al*., 2006). They also repress genes involved in cell wall loosening and organisation of the cytoskeleton, events crucial for endosperm weakening and radical extension during germination.

### Germination repression: the role of PIFs

The Phytochrome Interacting Factor (PIF) *PIL5* has two peaks of expression in both newly dispersed and overwintered seeds. *PIL5* upregulates the GA receptor *GID1A* and *ABI5* and downregulates *ABI4* and *CYP707A2* (Oh *et al*., 2009); expression patterns and correlation analysis of newly dispersed seeds (but not in overwintered seeds) support this for *GID1A* and *ABI5*. However, *PIL5* is not correlated with *ABI4* expression in spring-dispersed seeds and is positively correlated in overwintered seeds. Furthermore, *ABI4* – a repressor of *CYP707A2* expression (Shu *et al*., 2013) – is significantly positively correlated with this gene in both newly sown and overwintered seeds. There was also no correlation in either study between *PIL5* and the expression patterns of either *GA3ox1* or *GA2ox2*, which are negatively and positively regulated (respectively) by PIL5 (Oh *et al*., 2009). This highlights the complex system of positive and negative control operating over time in the soil seed bank that is not always revealed in mutant and single time-point laboratory studies.

### Germination derepression: the role of PIFs

The PIF family transcription factor *SPT*, is highly expressed in both studies when germination potential is highest. *SPT* has an important role in the regulation of primary dormancy and is reported to repress *ABI4*, *GA3ox1* and *MFT*, and induce *ABI5* expression (Penfield *et al*., 2005; Vaistij *et al*., 2013). Here we found that the correlation between *SPT* expression and these genes is more complex in the soil seed bank. *SPT* is positively correlated with *ABI4* in both newly dispersed and overwintered seeds (Table1; and Footitt *et al*., 2011). The same is true for *GA3ox1* in overwintered seeds, but not newly dispersed seeds. The correlation with *ABI5* is negative, but not significantly so. This again serves to emphasise the complex interactions in progress in the integration of the temporal and spatial sensing pathways that are influenced by seed history.

**Table 1:** Linear correlation coefficients of expression comparisons between *SPT*, *MFT* and selected genes in buried seeds recovered from the field

	Arabidopsis ecotype and burial cycle		
Cvi spring	Cvi autumn	Bur autumn
Gene correlations with *SPT*			
*ABI4*	0.949^b^	0.7182^b^	0.862^a^
*ABI5*	−0.592	−0.334	0.603^c^
*PYR1*	0.947^b^	0.600^c^	0.764^b^
*CYP707A2*	0.760^c^	0.722^b^	0.767^b^
*GA3ox1*	−0.249	0.727^b^	0.846^a^
*MFT*	−0.642	−0.850^a^	0.678^c^
Gene correlations with *MFT*			
*ABI3*	0.694^d^	0.731^b^	0.806^b^
*ABI5*	−0.001	0.229	0.732^c^
*RGA2*	−0.328	−0.681^c^	0.746^b^
*RGL2*	0.019	−0.728^b^	0.736^b^

Arabibopsis ecotype Cvi: Cvi Spring, seeds were buried in May 2008 with final recovery in October 2008. Cvi Autumn, seeds were buried in October 2007 with final recovery in October 2008. Arabibopsis ecotype Bur: Bur Autumn, seeds were buried October 2009 with the final recovery in October 2010. In order to consider only the response to seed burial, data from seeds before burial was omitted as their response is determined by prior maternal maturation conditions. Statistical significance: ^a^*P* < 0.001; ^b^*P* < 0.01; ^c^*P* < 0.05; ^d^*P* < 0.1. For Cvi spring data see Supporting Information Table S1, for Cvi and Bur autumn data see supplementary datasets S1 in Footitt *et al*. (2011, 2013).

This complex cycle of gene induction and repression by DELLA and PIF gene families is rapidly removed by interaction with the GID protein-GA complex and PHYB. The *PIF* family members are repressed by binding to RGL2 and RGA to form an inactive complex (Gallego-Bartolome *et al*., 2010). PIF proteins are released when the GID protein-GA complex binds DELLA proteins. This leads to DELLA degradation by the proteosome and the targeting of released PIL5 and SPT to the proteasome by PHYB in the light (Daviere *et al*., 2008); we show that this removes the final layer of dormancy resulting in germination. Phytochromes have an important role in the control of seasonal germination timing (Donohue *et al*., 2012). The magnitude of the transcriptional response to light is seen in *GA3ox1* where transcript amounts increased *c*. 600-fold when shallow dormant seeds were exposed to 5 min of red light (Cadman *et al*., 2006), whereas deeply dormant seeds are unresponsive to light potentially via the action of PHYA which has a strong dormancy-associated expression pattern (Footitt *et al*., 2013) and contributes to dormancy in cold-matured seeds (Donohue *et al*., 2008; Heschel *et al*., 2008; Dechaine *et al*., 2009).

### Maintaining shallow dormancy (ABA signalling pathway)

Underlying the regulation of germination potential is the operation of ABA signalling that operates via low-sensitivity (shallow dormancy) and high-sensitivity (deep dormancy) pathways (Footitt *et al*., 2011). From June to August dormancy is controlled by the low-sensitivity pathway after which dormancy rapidly increased in the field. During this period expression of genes for ABA catabolism *CYP707A2*, the ABA receptor *PYR1*, the repressor of ABA signalling *ABI2*, and the ABA-induced transcription factor *ABI4*, all decreased except when germination potential was high in August (Figs5). This is consistent with seeds overwintered in field soils where expression of these genes was also higher when germination potential was maximal in June–July (Footitt *et al*., 2011).

### Entry into deep dormancy

As autumn soil temperature decreased, the onset of deep dormancy occurred in newly dispersed and overwintered seeds. As dormancy (AR50 values) increased so did expression of the ABA receptor gene *PYL7* and the SNF1-related protein kinases, *SnrK 2.1* & *2.4*. The latter are positive regulators of ABA signalling acting downstream of ABA receptors to activate transcriptional regulators (Nambara *et al*., 2010). Expression of these SnrK2 protein kinases are inversely related to soil temperature in Cvi whereas in the Bur accession *SnrK 2.1* is inversely [ABA] and *SnrK 2.4* is positively related to temperature, indicating that as ABA does not increase as deep dormancy develops these genes may be central to controlling ABA sensitivity in response to seasonal changes in soil temperature (Footitt *et al*., 2011, 2013). Hormone synthesis responds to the changing conditions with a sustained increase in expression of *NCED6* (ABA biosynthesis) and *GA2ox2* (GA catabolism) in both newly dispersed and overwintered seeds consistent with increasing dormancy. At the same time both *ABI3* and *ABI5* increased consistent with their roles in blocking the seed/seedling transition (Nambara *et al*., 2010; Graeber *et al*., 2012).

In the work presented, analysis of the Arabidopsis winter annual ecotype Cvi introduced into the soil seed bank at their natural time of dispersal in spring has revealed their physiological and transcriptional response on introduction to a warming soil seed bank. In Fig.7 we contrast this to overwintered seeds of Cvi (Footitt *et al*., 2011) and seeds of the summer annual ecotype Bur (Footitt *et al*., 2013) to identify differences between seeds forming the short-term and persistent seed bank. The schematic illustrates how subtle differences in the temporal expression of key genes linked to temporal and spatial sensing may determine germination patterns resulting in winter and summer annual lifecycles. Seeds passing through the shallow dormancy cycle of the short-term seed bank (Cvi spring and Bur) show rapid responses to environmental change in the spatial sensing phase of the cycle manifested in rapid changes in germination potential. We suggest deep dormancy cycling in the persistent seed bank (overwintered Cvi) dampened these physiological responses through temporal sensing and thermal time measurement via *DOG1*. In each case seeds germinate when the thermal window permitting germination coincides with ambient temperature and spatial environmental signals (e.g. nitrate concentrations and light intensities). High seed sensitivity to these environmental signals (e.g. temperature, nitrate, light) occur when the expression of key repressing genes (e.g. *DOG1*, *CIPK23*, *PHYA*, respectively) are at their lowest. Subtle differences in the response of seeds from the persistent and short-term seed bank will contribute to bet hedging against variable soil conditions.

## Supporting Information

Table S1 QPCR primers

Table S2 Linear correlation coefficients for comparisons between environmental, physiological and molecular parameters for buried seeds
