# Supplementary material for: Environment sensing in spring-dispersed seeds of a winter annual Arabidopsis influences the regulation of dormancy to align germination potential with seasonal changes
Source: New Phytol. 2014 Jan 21;202(3):929–39. doi: 10.1111/nph.12694 (PMC4235297; doi:10.1111/nph.12694)
Supplement: Table S1 — QPCR primers [file nph0202-0929-SD1.doc]

**Supporting Information Table S1 QPCR primers.** Primer sets for genes used to evaluate the dormancy cycling behaviour of the Arabidopsis ecotype Cvi in the field.

| **Gene ID** | **Annotation** | **Forward primer** | **Reverse primer** |
| --- | --- | --- | --- |
| At5g57050 | *ABI2* | TTCAGAATCTCTAAATGGCTGAAAC | AAAAGGACTAAAATCAGAGTTTCCTG |
| At3g24650 | *ABI3* | CCTTGGAACAGGACGATGA | AAGAGAGGTTGGTGGTGGTG |
| At2g40220 | *ABI4* | GAGATCCGAGAGCCACGTAA | GGAAGAGGAGGTGGAAGGAG |
| At2g36270 | *ABI5* | TAATGGAGGAGGTGGTGGTG | CTGCTGCTTGTTGTTGATTGTT |
| At2g29090 | *CYP707A2* | CAATTCCTTCTTCGCCACTC | ATGGTATGGACCTTGGTGGA |
| At5g45830 | *DOG1* | GACGGCTACGAATCTTCAGG | AGCTAGCTGCTCCGCACTTA |
| At1g30040 | *Ga2ox2* | CTATTGGCTTCTTCTTCGGCTTG | AGTTCTTCGGCAACCATCTC |
| At1g15550 | *Ga3ox1* | GCTACCTGTCCAACGCAAG | CGAGCCAGTGATGGTGAA |
| At3g05120 | *GID 1A* | GCTGCGAGCGATGAAGTTA | AACCCATGTATTGAGAGGAACC |
| At1g18100 | *MFT* | GAGGCACAAATCCCTCAAGC | CCGAAAAGTACCAATATGTAACG |
| At3g24220 | *NCED6* | TTCAAGATACCGACACTTCCTG | GGCGATTCTGCTCCATAGG |
| At1g77760 | *NR1* | ATTCAGGCTTATACCACCAAGC | ACAATCGAGTCCTTCCTTCTCTC |
| At1g12110 | *NRT 1.1* | ACTGCGAACAAGCAAGTGG | ACCCGAAACCCGAGACAC |
| At2g20180 | *PIL5* | CGTCACGGAGAGGATAATGAAG | AAAACCAGAGACTTCACACCAAG |
| At4g01026 | *PYL7* | GTAAATGTCAAATCTGGTCTTCCA | TGCACAGTCAAGATCGAAGAGT |
| At4g17870 | *PYR1* | GACGATTCGACAAACCACAA | GCGCATCTCGAAGTTTTGTT |
| At1g14920 | *RGA2* | CGATTCGGCTTCTTCGTCTA | AGCATTTCAACCGCTTGTTT |
| At3g03450 | *RGL2* | CTGCGTTTCCAAAGGAAGAG | GTCGGATCCTCTTGCTGCTA |
| At5g08590 | *Snrk 2.1* | TCATCCGGTTCAAGGAGGT | CGGCAGTACAGATTCGATCA |
| At1g10940 | *Snrk 2.4* | ATCTGTGATTTTGGTTATTCCAAGT | AGAAAGGACCTCAGGTGCAA |
| At4g36930 | *SPT* | GATCTTTTAGGTCAGGTTGTCCATC | GTCATTCCAACGCAAACATAAC |
| At4g34270 | *TIP41-like* | GTGAAAACTGTTGGAGAGAAGCAA | TCAACTGGATACCCTTTCGCA |

*DOG1* universal primers for splice variants in Bentsink *et al*. (2006).
